# Novel Hybridized Computational Paradigms Integrated with Five Stand-Alone Algorithms for Clinical Prediction of HCV Status among Patients: A Data-Driven Technique

**DOI:** 10.3390/life13010079

**Published:** 2022-12-27

**Authors:** Zachariah Madaki, Nurettin Abacioglu, A. G. Usman, Neda Taner, Ahmet. O. Sehirli, S. I. Abba

**Affiliations:** 1 Department of Pharmacology, Faculty of Pharmacy, Near East University, North Cyprus, Mersin-10, 99138 Nicosia, Türkiye; 2 Operational Research Centre in Healthcare, Near East University, North Cyprus, Mersin-10, 99138 Nicosia, Türkiye; 3 Department of Analytical Chemistry, Faculty of Pharmacy, Near East University, North Cyprus, Mersin-10, 99138 Nicosia, Türkiye; 4 Department of Clinical Pharmacy, Faculty of Pharmacy, Istanbul Medipol University, 34810 Istanbul, Türkiye; 5 Department of Pharmacology, Faculty of Dentistry, Nicosia, Near East University, North Cyprus, Mersin-10, 99138 Nicosia, Türkiye; 6 Interdisciplinary Research Centre for Membrane and Water Security, Faculty of Petroleum and Minerals, King Fahd University, Dhahran 31261, Saudi Arabia

**Keywords:** hepatitis C status, machine learning, artificial intelligence, clinical variables, hybrid paradigms

## Abstract

The emergence of health informatics opens new opportunities and doors for different disease diagnoses. The current work proposed the implementation of five different stand-alone techniques coupled with four different novel hybridized paradigms for the clinical prediction of hepatitis C status among patients, using both sociodemographic and clinical input variables. Both the visualized and quantitative performances of the stand-alone algorithms present the capability of the Gaussian process regression (GPR), Generalized neural network (GRNN), and Interactive linear regression (ILR) over the Support Vector Regression (SVR) and Adaptive neuro-fuzzy inference system (ANFIS) models. Hence, due to the lower performance of the stand-alone algorithms at a certain point, four different novel hybrid data intelligent algorithms were proposed, including: interactive linear regression-Gaussian process regression (ILR-GPR), interactive linear regression-generalized neural network (ILR-GRNN), interactive linear regression-Support Vector Regression (ILR-SVR), and interactive linear regression-adaptive neuro-fuzzy inference system (ILR-ANFIS), to boost the prediction accuracy of the stand-alone techniques in the clinical prediction of hepatitis C among patients. Based on the quantitative prediction skills presented by the novel hybridized paradigms, the proposed techniques were able to enhance the performance efficiency of the single paradigms up to 44% and 45% in the calibration and validation phases, respectively.

## 1. Introduction

Liver diseases are mostly caused by hepatitis C virus (HCV) infections worldwide. HCV is mostly transmitted prenatally, sexually, and parenterally. This virus equally induces immune-mediated chronic and acute necroinflammatory liver diseases, which, in turn, makes HCV account for about 60–80% of chronic hepatitis in infected adults, with a relatively high clearance rate, which is reported in most African countries [1]. Owing to the fact that both the vaccine and the cure for most hepatitis, such as hepatitis B, is of paramount significance, the study of various components of the successful immune responses, strategies of viral immune evasion, as well as the disease pathogenesis mechanisms are necessary [1]. Recently, the understanding of HCV infection at the early stage has been achieved based on a study conducted on chimpanzees, which are considered to be the only animal that can be infected with HCV and HBV at the same time. Moreover, a strategy based on an infectious molecular clone, which is related with the HCV GB virus isolated from GB patients, as well as infectious to tamarins, has been developed recently in order to provide an in vivo surrogate model for HCV, and hence will aid in the understanding of its mechanism effectively. Furthermore, in view of in vivo analysis of this virus, different experimental techniques are readily available recently, which enable the elucidation of HCV replication as well as the processing polyprotein infection of hepatoma and the neutralization of pseudotype-particle infections by antibodies [2].

In addition, the computational health informatics (CHI) is a branch of medical informatics that couples the application of artificial intelligence (AI) and medical science towards understanding and the elucidation of various diseases, viral routes, bacterial infection mechanisms, and their medical complications [3]. Moreover, CHI is seldom referred to as clinical AI, which subsists within the interface of health informatics and machine learning (ML). Furthermore, the implementation of CHI in understanding the mechanisms and behaviours of HCV is of paramount importance [4]. Hence, various studies have reported the application of ML in the predicting, modelling, simulation and estimation of HCV. For instance, Haga et al. [5] employ the applications of various AI models for identifying HCV variant resistance through whole genome sequencing. Based on the performance of both the training and validation datasets, the support vector regression (SVR) depicts higher prediction performance than other AI models used in the study. Moreover, Robert et al. [6] reported the application of multivariate techniques in modelling HCV/HIV infected patients using liver biopsy. The performance results of the techniques indicate the feasibility of the application of these techniques in understanding HIV and HCV. Recently, Safdari et al. [7] reported the feasibility of implementing different ML classifiers for classifying patients as positive and negative based on the presence of HCV in the subjects’ blood serum. The obtained results indicates the feasibility of using ML in classifying the patients’ statuses based on the training data.

**Figure 1:**
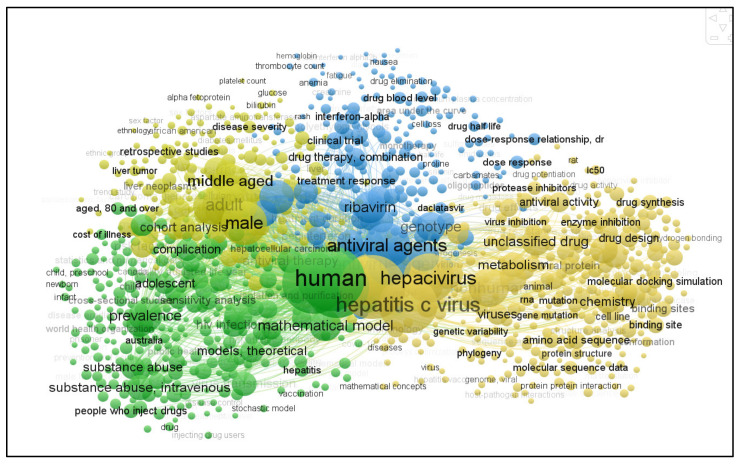
A scan of 1142 document results from the published technical literature based on major keywords used in HCV modelling (source, Scopus 2022).

Furthermore, a scan of 1142 document results (see Figure 1) demonstrates the major keywords used in the literature regarding HCV modelling. The results demonstrate that there is a need for work on CHI for HCV elucidation, owing to its simplicity, quickness, and cost-effectiveness.

Recently, computational approaches play a significant role, with remarkable capability in simulation and modelling of highly chaotic medical processes and phenomena. Indeed, ML depicts a promising ability in managing large amounts of non-linear, complex, and complicated datasets. A large number of articles have been developed using various ML techniques in order to demonstrate the success of various ML predictive skills in CHI and bioinformatics [8,9,10]. Hence, artificial neural network (ANN) and SVR have emerged as the most frequently and widely employed techniques among the ML techniques in bioinformatics in recent years. Their increasing popularity may be attributed to their promising performance, as well as to their robustness in modelling highly stochastic and complex medical patterns [11]. In addition, other advanced techniques, such as polysomnography (PSG), which is considered as a gold standard tool for diagnosing sleep apnea (SA), can equally be used as a reliable computational tool for diagnostics [12]. Moreover, Chuma et al., reported the application of a convolutional neural network (CNN) combined with other sensors to create a new solution to fight COVID-19 transmission. The findings of their study demonstrates that the proposed method has a cough detection test accuracy of 88.0% using Alex Net CNN with people 1 m away from the microwave radar sensor; a test accuracy of 80.0% with people 3 m away from the radar sensor; and test accuracy of 86.5% with a single mixed dataset with people 1 m and 3 m away from the radar sensor [13]. More information can equally be found in [14].

Based on the above-mentioned literature, it is clear that the implementation of ML in various medical studies is significant, for example, in understanding the HCV status of a patient. Nonetheless, the predictive performance generated by some of these techniques still encountered different inadequacies, imprecision, and a lack of accuracy, especially in the presence of highly non-stationary and chaotic medical datasets, despite the robust merits of the ML techniques. In this kind of scenario, this kind of single model could not meet the needed outcomes, especially if there is no strong pre-processing technique before starting the simulation [11]. Therefore, it is necessary, in order to overcome the above mentioned drawbacks, to develop a well-trained novel hybridized technique that will couple the interactive linear regression (ILR) and the ML approaches. Based on Zang [15], the major concept regarding model hybridization in different fields is to improve the prediction performance of the existing single models, which is possible, owing to the fact that each approach has its own peculiar, exceptional and unique features with quite different prediction patterns. The implementation of ILR in bioinformatics was first introduced in the current study as a linear prediction technique. Moreover, the integration of ILR with SVR, generalised neural network (GRNN), Gaussian process regression (GPR), and adaptive neuro-fuzzy inference system (ANFIS) was proposed in order to enjoy the merits of the prime strengths of ILR and the non-linear techniques. Based on the previous research conducted in the published literature, to the best knowledge of the researchers of the current work, no study thus far has reported on the integration of ILR technique with non-linear approaches in modelling HCV.

The major motivation of this study is to integrate the abilities of various rugged AI-based techniques including; SVR, GRNN, ANFIS, GPR, and ILR linear technique for the simulation of patients’ HCV infection statuses. Before the modelling step, using the single models, the descriptive statistics and correlational matrix were conducted to understand the behaviour and relationship between the variables. Moreover, the second step proposes hybrid learning techniques that integrate both the ILR linear model and the AI-based techniques (ILR-SVR, ILR-GRNN, ILR-ANFIS and ILR-ANFIS) to take advantage of both non-linear and linear patterns of the approaches. Hence, both empirical and theoretical findings indicate that coupling different techniques can serve as an efficient and effective method of improving performance prediction of different parameters [15].

## 2. Materials and Methods

### 2.1. Proposed Model Development Method

The need for understanding the science and knowledge of data is of paramount importance recently in medical informatics, CHI, and bioinformatics. The data used in the current study contains 615 observations with 13 attributes of the blood donors, subjects, and the hepatitis C patient’s laboratory, as well as demographic variables, which are composed of: age, sex, albumin (ALB), alkaline phosphatase (ALP), alanine transaminase (ALT), aspartate aminotransferase (AST), basal insulin level (BIL), cholinesterase (ChE), cholesterol (CHOL), creatinine (CREA), gamma-glutamyl transferase (GGT) and protein (PROT). In addition, the dataset is equally obtained from UCI Machine Learning, and for more detailed information, it can be found at https://archive.ics.uci.edu/ml/datasets/HCV+data, accessed on 6 October 2022. Moreover, five different single models informing on GPR, SVR, ANFIS, GRNN, and ILR were used in predicting the hepatitis status. These techniques were composed of four non-linear (GPR, SVR, ANFIS, and GRNN) and one classical linear method to inform of ILR. Furthermore, based on different researches depicted in the recent technical literature for simulation, modelling, prediction and forecasting, the AI-based models usually demonstrate higher performance than the classical linear method, as demonstrated by [16,17,18,19].

Moreover, based on the limitations of the single data-driven approaches in capturing the HCV status, four different novel hybrid techniques, namely, the ILR-SVR, ILR-ANFIS, ILR-GRNN, and ILR-GPR techniques, were proposed to improve and boost the performance of the single models.

### 2.2. Gaussian Process Regression (GPR)

This technique is considered to be a robust non-linear regression approach, prediction, nonparametric, probabilistic, unsupervised and supervised learning technique, which generalizes the complex and non-linear function mapping within the hidden function of the datasets. In recent years, GPR is obtaining more popularity in different fields related to computational techniques. GPR has the ability to handle highly stochastic non-linear behaviour due to its use of kernel functions. Furthermore, another advantage of a GPR model is that it can provide a satisfactory response to the training data.

### 2.3. Support Vector Regression (SVR)

**Figure 2:**
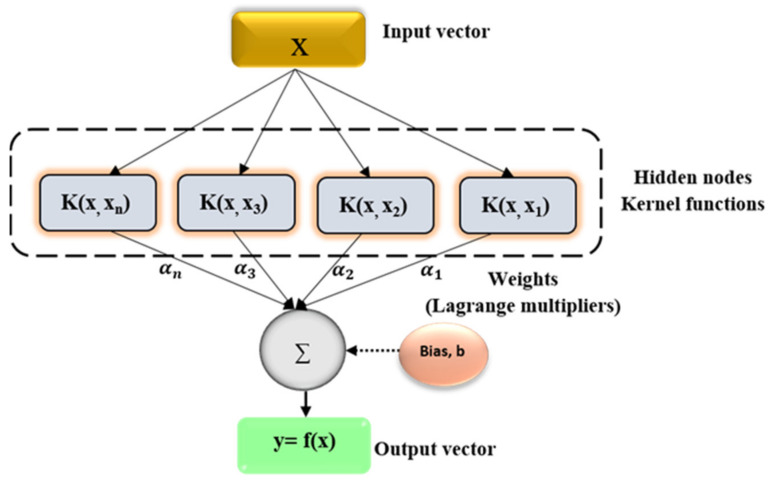
Structure of the SVR model.

This model was developed based on the concept of support vector machine (SVM), which is generally used in solving problems through regression and classification approaches [9,20,21,22,23]. SVR is an established computational technique with various merits, such as good noise-toleration, superior generalization ability, and high learning speed [24]. Generally, the input variables from the datasets were mapped into a high dimensional feature filter architecture via a kernel operator using the SVR [24,25,26]. This regression technique has the ability of converting a non-linear problem into a linear problem via understanding the learning complexity of the relationship between the input and output variables [27,28]. The structure of SVM can be found in Figure 2.

### 2.4. Interactive Linear Regression (ILR)

In general, linear regression (LR) is one of the most widely used computational methods for modelling a wide range of input and output variables. It is worth noting that there is a link connecting both the simple and the complex variables when it comes to determining the best combination of parameters for the best prediction efficiency, which is tied to the output variable [29]. Systematic regression has been defined by several modellers as an advanced option that uses the best set of input data by eliminating or inserting variables under the impact of the residual sum of the squares [30].

By examining the effects of the variables, the ILR adheres to their consistent variations. Each factor that fails to make contributions to and meet the model’s procedure might be removed, one by one, to minimize their influences [31]. The principle of ILR might be shown using MLR [32,33,34,35,36]. The method of integrating or interacting a fixed input from LR is known as interactive regression [37].

### 2.5. Generalized Regression Neural Network (GRNN)

The generalized regression neural network (GRNN), also known as the lazy training method model, was developed by Specht [38] to behave in the manner of a regression method for generating a relationship between the independent variable (X) and the dependent variable (Y) with a nonlinear regression estimation for a smaller group of data. The input layer is similar to that of a conventional neural network, in that its main purpose is to train the input data, and the size of the input vectors is the main determinant of the number of neurons required for training. The model training begins immediately in the pattern layer due to the Gaussian kernel’s conversion of previously input data. The smoothing parameter (σ) is used to calculate the weight of each neuron in this layer. This parameter is known as the “hyper-parameter of the GRNN model,” and it contributes to the GRNN model’s prediction accuracy [39]. Its general form is depicted below.

(1) $$P_{i}=exp\left(-\frac{{\left(X-X_{i}\right)}^{T}\left(X-X_{i}\right)}{2\sigma^{2}}\right)$$

where X is the input data of the testing dataset, $X_{i}$ is the *i*th input of training dataset, and σ is the smoothing parameter.

### 2.6. Adaptive Neuro-Fuzzy Inference System (ANFIS)

Adaptive Neuro-Fuzzy Inference System (ANFIS) is an integrated tool that uses the fuzzy Sugeno model approach that derives the benefit of both ANN and fuzzy logic in a single framework. Recently, ANFIS has been utilized in predicting and modelling complex datasets, such as in hydrological applications and wastewater modelling [40]. ANFIS has the ability to approximate the real functions, and therefore is regarded as the real-world estimator [41]. Fuzzy logic involves transforming input data values into fuzzy values through the application of membership functions (MFs). The values range between 0 and 1. Likewise, in ANFIS, model nodes work as MFs, as well as permit the modelling between the relations of the input and the output.

Assume the FIS contains two inputs, ‘x’ and ‘y’, and one output, ‘f’; a first order Sugeno fuzzy has following rules.

(2) $$\mathrm{Rule}\;1\;\mathrm{if}:\mu \left(x\right)\;\mathrm{is}\;A_{1}\;\mathrm{and}\;\mu \left(y\right){\;B}_{1}{\;\mathrm{then}\;f}_{1}={\;p}_{1}x+q_{1}y+r_{1}$$

(3) $$\mathrm{Rule}\;2\;\mathrm{if}:\mu \left(x\right)\;\mathrm{is}\;A_{2}\;\mathrm{and}\;\mu \left(y\right){\;\mathrm{is}\;B}_{2}{\;\mathrm{then}\;f}_{2}={\;p}_{2}x+q_{2}y+r_{2}$$

$A_{1},{\;B}_{1},\;A_{2},\;B_{2}$ parameters are membership functions for x and y inputs, and $p_{1},\;q_{1},\;r_{1},{\;p}_{2},\;q_{2},\;r_{2},$ are the outlet function’s parameters. The structure and formulation of ANFIS follow a five-layer neural network arrangement. For more detail of the ANFIS model refer to [40,42,43].

### 2.7. Hybrid Techniques Development

The predictive efficiency of various computational techniques is generally linked with different factors, such as time scaling, determination of the input and output variables, and the model setup. All of these depict an impact on the overall performance of the computational technique. Therefore, AI-based drawbacks could be managed by exploring unique approaches that capture both simple linear and complex non-linear connections among the input-output variables. As indicated in the previous chapter, ML algorithms such as ANFIS, GRNN, SVR, and GPR depict a promising ability in various computational fields. Based on Olson et al. [9], both the non-linear techniques (such as ANFIS, GRNN, SVR, and GPR) and the linear approaches (such as MLR, SWLR, ILR, RLR, and MVR) have shown reliable prediction performances. Nevertheless, combining the two approaches in a highly stochastic and complex dataset will result in enjoying the benefits of the two domains, which in turn lead to the boosting of performance efficiency of the single models. Moreover, the “no free lunch” theory suggested that there is no single technique that can be used in all kinds of datasets, due to the fact that the performance efficiency of any model is heavily influenced by data qualities such as linearity, size, and normality. Aside from that, several types of research have demonstrated that effectiveness indexes for distinct models might fluctuate even when using the same dataset [44,45,46,47,48]. As a result, five different single data-intelligence techniques are used in modelling the hepatitis status: ANFIS, GRNN, SVR, GPR, and ILR in the first scenario. Moreover, the second scenario involves the boosting of the first scenario performance through integrating the linear ILR and the non-linear techniques (ILR, ANFIS, GRNN, SVR, and GPR) to benefit the advantage of the remarkable features and strengths of both ILR and non-linear approaches in predicting multiple data patterns.

### 2.8. Performance Objectives

The use of statistical metrics for evaluating the performance objectives of the techniques/models used in any computational approach is of paramount importance. These performance objectives mostly checked the performance of the models based on the comparative performance of the simulated and observed clinical values. In the current study, we employed two statistical fitness determinants, namely: determination co-efficient (*DC*) and Pearson correlation co-efficient (*PCC*), and two statistical error indices in the forms of mean-squared error (*MSE*) and root-mean-squared error (*RMSE*).


(4) $$MSE=\frac{1}{N}\overset{N}{\underset{i=1}{\sum }}{\left(HS_{\left(p\right)}-HS_{\left(o\right)}\right)}^{2}$$

(5) $$RMSE=\sqrt{\frac{1}{N}\overset{N}{\underset{i=1}{\sum }}{\left(HS_{\left(p\right)}-\;HS_{\left(o\right)}\right)}^{2}}$$

(6) $$DC=1-\frac{\sum_{i=1}^{N}{\left(HS_{\left(p\right)}-\;HS_{\left(o\right)}\right)}^{2}}{\sum_{i=1}^{N}{\left(HS_{\left(p\right)}-\;HS\prime_{\left(o\right)}\right)}^{2}}$$

(7) $$PCC=\frac{\sum_{i=1}^{N}\left[HS_{\left(t\right),i}-\bar{HS_{\left(t\right)}}\right]\left[{\hat{HS}}_{\left(t\right),i}-{\tilde{HS}}_{\left(t\right)}\right]}{\sqrt{\sum_{i=1}^{N}{\left[HS-HS_{\left(t\right)}\right]}^{2}{\left[{\tilde{HS}}_{\left(t\right)}-{\tilde{HS}}_{\left(t\right)}\right]}^{2}}}$$

### 2.9. Description of the Data Set and Model Validation

The basic goal of good data-driven techniques is for the system to give a collection of data from the indicators in use as a basis for correctly predicting unknown variables [49,50,51]. The data used in the current study contains 615 observations and with 13 attributes of the blood donor subjects and the hepatitis C patient’s laboratory, as well as demographic variables, which are composed of: age, sex, ALB, ALP, ALT, AST, BIL, ChE, CHOL, CREA, GGT, and PROT, as mentioned in the previous chapter. In addition, the dataset is equally obtained from UCI Machine Learning, and for more detailed information, it can be found at https://archive.ics.uci.edu/ml/datasets/HCV+data, accessed on 6 October 2022.

Moreover, consider the fact that various constraints, such as overfitting, result in good training results that may not always match the testing results. Validation techniques include k-fold cross-validation, holdout, leaving one out, and others. The fact that the evaluation and training datasets are independent is the most significant advantage of the k-fold cross-validation procedure [52,53,54,55,56,57].

According to the k-fold cross-validation, the data have been divided into 65% for the calibration stage and 35% for the validation stage in this study. As a result, it is critical to understand that various validation models might be used for data testing [58,59,60,61,62].

Moreover, regarding the imbalance in informatics analysis, actual case validation can be solved via data elaboration from experiments or clinical findings using an exploratory analysis method in addition to the internal and external validation methods employed in the current study. Moreover, other simulation techniques, such as standardization, outliers sieving, normalization, and feature scaling can be employed in addressing these drawbacks. Furthermore, electronic health record data, which is expanding to support quality improvement and research, can be used; however, this requires standardization of the data and validation within and across organizations. Information models (IMs) are created to standardize data elements into a logical organization that includes data elements, definitions, data types, values, and relationships. To be generalizable, these models need to be validated across organizations using different internal and external validation processes, such as the k-fold cross-validation, which is employed in the current study.

The structures of the techniques as well as their properties indicating the comparative advantages and disadvantages of the simulation process are indicated in Table 1.

## 3. Results

**Figure 3:**
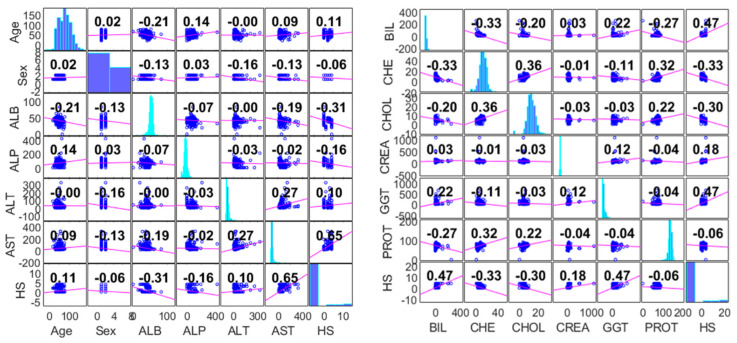
Correlation matrix of the input–output for the first 6–6 parameters.

Computational techniques used in CHI and medical informatics such as AI are regarded as robust and complex paradigms in recent times, owing to the fact that they have the ability to integrate more variables, which can be employed in fine-tuning the models. Hence, the more the complexity, the higher the uncertainty of the models’ outcomes. An exploratory technique based on a correlation matrix is presented in Figure 3, which indicates the relation and connection among the variables used in the current research, especially the input–output variables.

According to the correlation matrix shown in Figure 3, it can be seen that AST showed a strong correlation against the target hepatitis status, with the PCC-value equal to 0.65, while BIL and GGT showed relatively intermediate relation with the target, with both PCC values equal to 0.47. Moreover, all the other input variables depict a weak correlation, with PCC-value <0.4.

The current section presents the quantitative and graphical performance of the single models employed in the first scenario. According to Nourani et al. [63] for a model to be accepted as a reliable computational tool, it should have a minimum DC-value of 0.8 in both phases.

**Figure 4:**
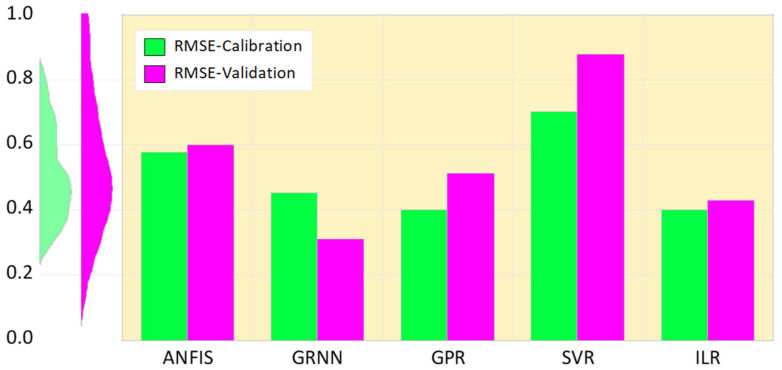
Comparative error performance of the single paradigms based on their RMSE.

Figure 4 represents the graphical comparative performance of the models based on their respective RMSE values. It is noteworthy that the higher the error values, the lower the performance of the models, and vice-versa.

## 4. Discussion

The quantitative performance of the models demonstrates that the non-linear techniques GRNN and GPR, as well as the linear ILR approach, were able to predict the hepatitis C status of the patients with a minimum DC-value of 0.8 in both the calibration and validation stages, while the ANFIS and SVR methods showed a DC-value > 0.8. This indicates that the GRNN, GPR, and ILR models has fulfilled the minimum requirements of HCV prediction from the patients’ blood serum, while ANFIS and SVR failed. The success of the ILR technique, even though it is a linear approach, is not surprising, owing to its high computational robustness which involves complex interaction between the input–output variables as compared with other classical linear techniques, such as MLR and SWLR. Furthermore, the predictive skills of the techniques can be visualized in order to graphically compare the performance of the techniques.

**Figure 5:**
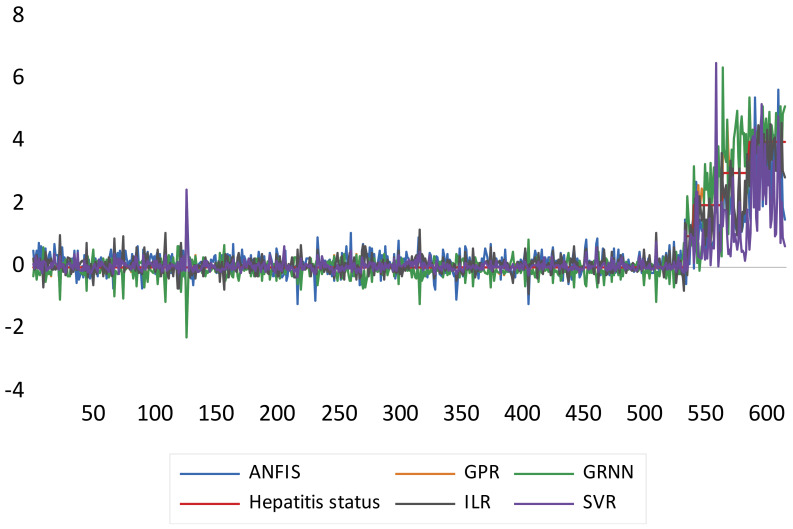
Response plot performance of the single paradigms.

**Figure 6:**
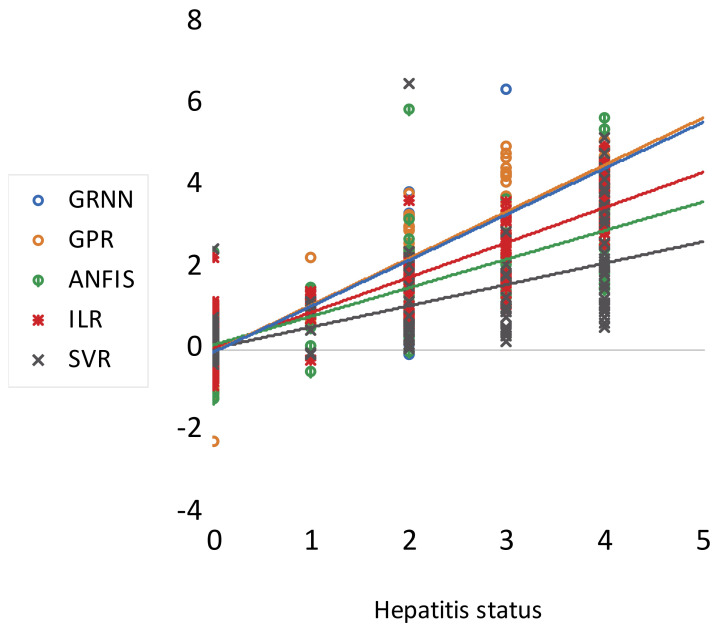
Scatter plot performance of the single paradigms.

Based on the graphical performance, indicated in Figure 4, it can be understood that ILR, GPR, and GRNN demonstrated higher performance accuracy compared to SVR and ANFIS. Moreover, more information regarding RMSE metrics can be found in [64,65]. Furthermore, the fitness of the techniques can equally be graphically compared using both a scatter plot and a response plot (see Figure 5 and Figure 6). Based on Figure 5 and Figure 6, the goodness-of-fits indicated in both the response and scatter plot performance are in line with the results demonstrated in Table 2. The performance skills of the single paradigms shown in the first scenario, in terms of quantitative and visualization format, depicts that the models failed at a certain stage in modelling the patients’ HCV statuses. Therefore, this led to the development of novel hybridized paradigms by coupling the linear and non-linear behaviour of the single paradigms in order to capture and simulate the complex behaviour of the hepatitis C status.

**Figure 7:**
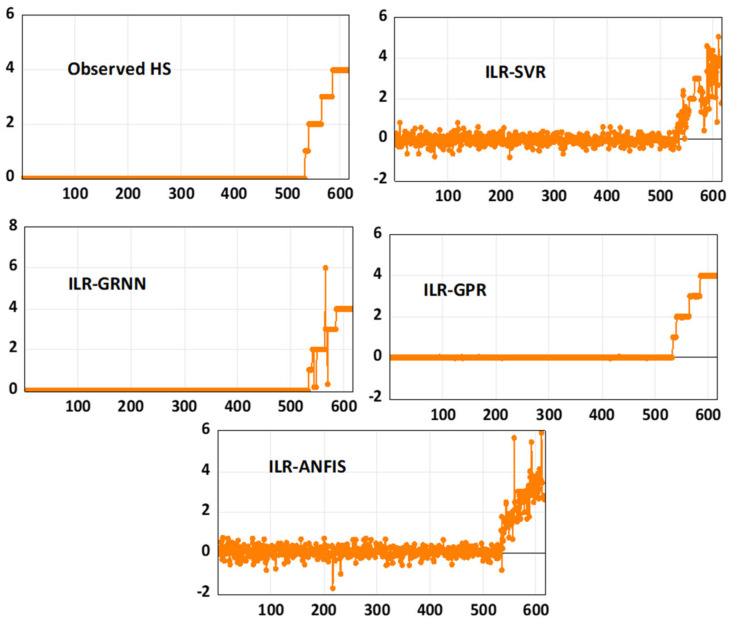
Response plot performance of the novel hybrid paradigms.

**Figure 8:**
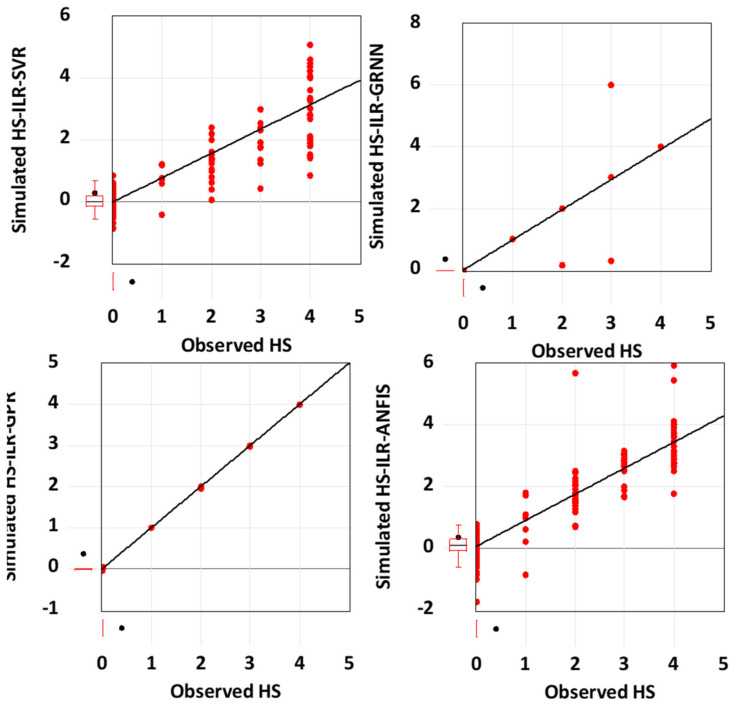
Scatter plot performance of the novel hybrid paradigms.

According to the prediction skills presented by the novel hybridized paradigms shown in Table 3, it can be seen that the proposed techniques were able to enhance the performance efficiency of the single paradigms up to 44% and 45% in the calibration and validation phases, respectively. Though all of the four proposed paradigms were able to predict the highly stochastic, complex, and chaotic HCV data in both the calibration and validation stages, the ILR-GPR and ILR-GRNN showed superior performance to the ILR-ANFIS and ILR-SVR, as shown in Figure 7 and Figure 8, respectively.

**Figure 9:**
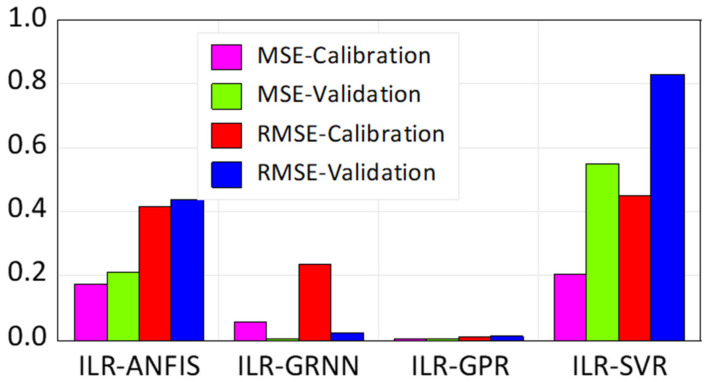
Comparative error performance of the novel hybridized paradigms based on their RMSE and MSE-values.

**Figure 10:**
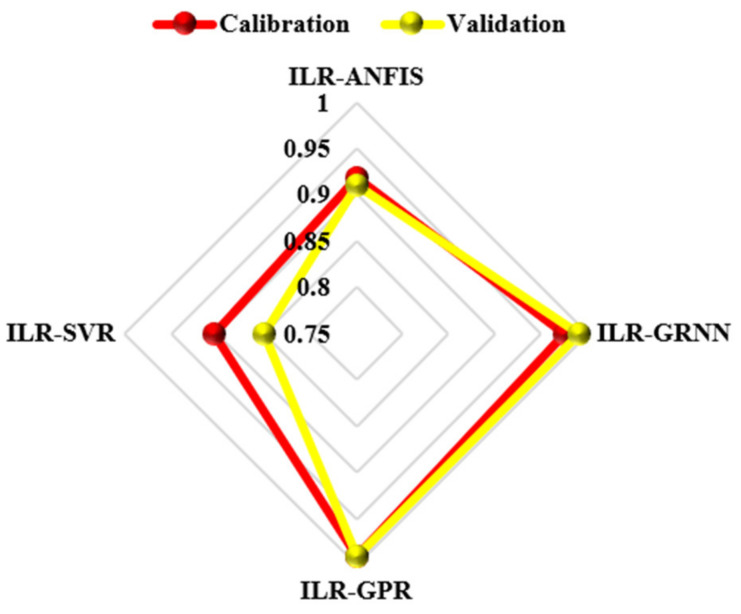
Comparative PCC-values using radar plot of the novel hybridized paradigms.

The visualizations presented in both Figure 7 and Figure 8, regarding the graphical goodness-of-fits of the novel hybridized paradigms, demonstrate superior and improved performance over the single paradigms. Moreover, the performance skills of the models can equally be compared based on their respective error performances and therefore inform of RMSE and MSE statistical metrics, as shown in Figure 9. Moreover, the performance of the models can be compared based on their PCC metrics in both the calibration and validation phases, respectively (see Figure 10).

**Figure 11:**
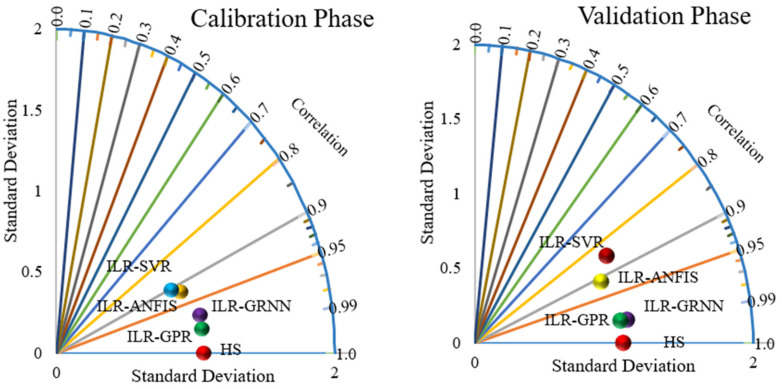
Taylor diagram performance of the novel hybridized techniques in both the calibration and validation steps.

Moreover, the performance of the novel hybrid algorithms can be presented using a new two-dimensional visualization called the Taylor diagram (see Figure 11). The Taylor diagram is used in presenting the performance metrics of various computational approaches in two-dimensional patterns, using different objectives such as RMSE, MSE, PCC, DC, mean absolute percentage error (MAPE), mean absolute error (MAE), kurtosis, standard deviation, skewness, etc. The current study employs the use of PCC against the standard deviation to indicate the performance skills of the novel hybridized data intelligent algorithms, as in presented in Figure 11. The visualizations demonstrated from Figure 7, Figure 8, Figure 9, Figure 10 and Figure 11 depict an improved performance of the novel hybridized algorithms over the single paradigms.

## 5. Conclusions

Health informatics is an emerging area of study that involves an intersection between different domains, such as epidemiology, health care, computer information systems and bioinformatics, which is mostly applied in early diagnosis of different deadly diseases as well providing an alternative medication to the patients. This novel technique is regarded as a cost-effective approach that employs minimum or fewer resources, which can help medical doctors, policy makers, and other health professionals in decision-making. Therefore, it is of paramount importance to apply these emerging computational techniques such as ML, AI, and metaheuristic approaches in diagnosing various diseases such as HBV and HCV infections, owing to the fact that these techniques were not well established in the technical literature. Among the major motivations of this work is the implementation of multi-model single paradigms, coupled with novel hybridized data intelligent algorithms, for hepatitis status modelling of various patients. Moreover, the overall findings of the current study can be summarized as follows:

1. Based on the correlation matrix result, AST showed a strong correlation against the target hepatitis status, with a PCC-value equal to 0.65, while BIL and GGT showed relatively intermediate relation with the target, with both PCC values equal to 0.47. Moreover, all the other input variables depicts a weak correlation with PCC-value <0.4.
2. The quantitative performance of the models demonstrates that the non-linear techniques GRNN and GPR, as well as the linear ILR approach, were able to predict the hepatitis C status of the patients with a minimum DC-value of 0.8 in both the calibration and validation stages, while the ANFIS and SVR methods showed a DC-value lower than 0.8. This indicates that the GRNN, GPR, and ILR models have fulfilled the minimum requirements of HCV prediction from the patients’ blood serum, while ANFIS and SVR failed.
3. The performance skills of the single paradigms shown in the first scenario in terms of quantitative and visualization formats depict that the models failed at a certain stage in modelling the patients’ HCV statuses. Therefore, this led to the development of novel hybridized paradigms by coupling the linear and non-linear behavior of the single paradigms in order to capture and simulate the complex behavior of the hepatitis C status.
4. Based on the quantitative prediction skills presented by the novel hybridized paradigms, it can be seen that the proposed techniques were able to enhance the performance efficiency of the single paradigms up to 44% and 45% in the calibration and validation phases, respectively.
5. The findings of the study also recommend and open a new door for the applications of recent and robust techniques, such as non-linear ensemble paradigms and metaheuristic approaches, for the prediction of hepatitis C status.

## Figures and Tables

**Table 1:** Computational performance power for the parameters used in the algorithms.

GRNN	GPR	SVR	ILR
Network type: Generalized regression	Cross Validation: 10-folds	Cross Validation: 10-folds	Cross Validation: 5-folds
Spread Constant: 1.0	Regression Learner View: Exponential GPR	Regression Learner View: Medium Gaussian SVR	Regression Learner View: Interaction linear
Epoch: 200 iterations	Feature selection: PCA deactivated	Feature selection: PCA deactivated	Feature selection: PCA deactivated
Learning time: 0.000001			
Training: Levenberg–Marquardt			
Validation checks: 8			

**Table 2:** Results of the ANFIS, GRNN, SVR, GPR and ILR models.

		Calibration		
Models	DC	PCC	RMSE	MSE
ANFIS	0.70	0.84	0.58	0.33
GRNN	0.82	0.90	0.45	0.20
GPR	0.86	0.93	0.40	0.16
SVR	0.55	0.74	0.70	0.49
ILR	0.86	0.92	0.40	0.16
		**Validation**		
ANFIS	0.69	0.82	0.60	0.34
GRNN	0.92	0.95	0.31	0.11
GPR	0.81	0.89	0.51	0.27
SVR	0.53	0.69	0.88	0.53
ILR	0.81	0.87	0.43	0.19

**Table 3:** Results of the hybridized paradigms for hepatitis C status modelling.

		Calibration		
Techniques	DC	PCC	RMSE	MSE
ILR-ANFIS	0.84	0.92	0.42	0.17
ILR-GRNN	0.95	0.98	0.23	0.05
ILR-GPR	0.99	0.99	0.00662	0.00004
ILR-SVR	0.82	0.90	0.45	0.20
		**Validation**		
ILR-ANFIS	0.83	0.91	0.44	0.21
ILR-GRNN	0.96	0.99	0.02	0.00
ILR-GPR	0.98	0.99	0.01	0.00006
ILR-SVR	0.80	0.85	0.83	0.55

## Data Availability

Not applicable.
